# Use of Emulsion-Templated, Highly Porous Polyelectrolytes for In Vitro Germination of Chickpea Embryos: a New Substrate for Soilless Cultivation

**DOI:** 10.1021/acs.biomac.2c00593

**Published:** 2022-07-08

**Authors:** Janja Majer Kovačič, Terezija Ciringer, Jana Ambrožič-Dolinšek, Sebastijan Kovačič

**Affiliations:** † Faculty of Natural Sciences and Mathematics, University of Maribor, Koroška 160, 2000 Maribor, Slovenia; ‡ Faculty of Education, University of Maribor, Koroška Cesta 160, 2000 Maribor, Slovenia; § Faculty of Agriculture and Life Sciences, University of Maribor, Pivola 10, 2311 Hoče, Slovenia; ∥ Department of Polymer Chemistry and Technology, National Institute of Chemistry, Hajdrihova 19, 1000 Ljubljana, Slovenia

## Abstract

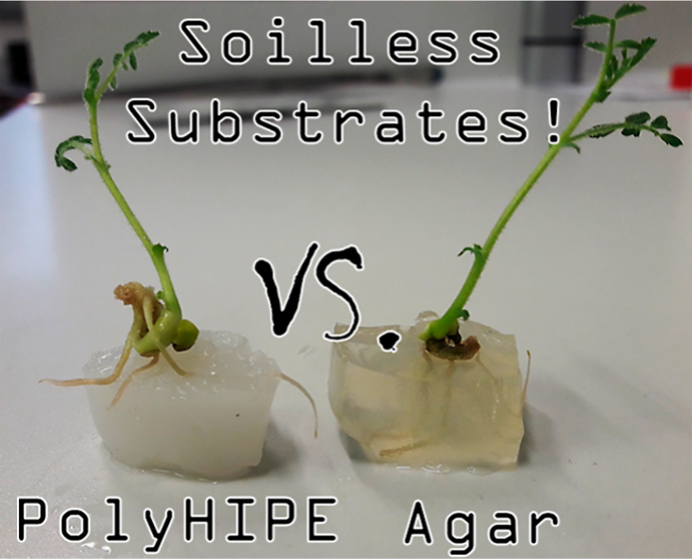

The application of highly porous and 3D interconnected microcellular polyelectrolyte polyHIPE (PE-PH) monoliths based on (3-acrylamidopropyl)-trimethylammonium chloride as soilless cultivation substrates for in vitro embryo culture is discussed. The embryo axes isolated from chickpea seeds are inoculated onto the surface of the monoliths and allowed to germinate. Germination study show that the newly disclosed PE-PH substrate performs much better than the conventionally used agar as the germination percentage, shoot and root length, fresh and dry weight as well as the number of leaves are enhanced. The PE-PHs exhibit a higher absorption capacity of the plant growth medium, that is, 36 g·g^–1^ compared to agar, that is, 20 g·g^–1^, and also survive autoclaving conditions without failing. The key advantage over standard agar substrates is that they can be reused several times and also without prior sterilization. These results suggest that PE-PHs with exceptional absorption/retention properties and robustness have great potential as soilless substrates for in vitro plant cultivation.

## 1 Introduction

Polyelectrolyte polymerized high internal phase emulsions, referred to as “polyelectrolyte polyHIPEs (PE-PHs),” are highly porous hydrogels with positive and/or negative charges distributed throughout the entire macromolecular network.^1^ Combining the properties of polyelectrolyte hydrogels and the microstructure of polyHIPEs, the PE-PHs are both superabsorbent and mechanically robust.^2,3^ A route that provides direct access to PE-PHs uses polymerization of ionic monomers within the external phase of an oil-in-water high internal phase emulsions (HIPEs). Presently, several PE-PHs are known such based on 2-acrylamido-2-methyl-1-propanesulfonic acid (AMPS),^4^ (3-acrylamidopropyl)-trimethylammonium chloride,^5^*N*-(3-sulfopropyl)-*N*-(methacryloxyethyl)-*N*,*N*-dimethylammonium betaine,^6^ [2-(methacryloyloxy) ethyl] trimethylammonium chloride),^7^ (vinylbenzyl)trimethylammonium chloride,^8^ styrene sulfonate,^9^ or based on copolymers containing sodium acrylate,^10^ methacrylic acid,^11^ Pluronic F-127 dimethacrylate,^12,13^ and AMPS.^14−16^ Some of these PE-PHs exhibited extraordinary capacities for the liquid absorption, for example, water uptakes of up to 980 g·g^–1^^17^ and are able to remove contaminants from water quickly and efficiently.^4,5,15,18^ This unusually high and rapid liquid absorption in PE-PHs is significantly higher than that of commercially available superabsorbent polymers, which absorb about 100 g·g^–1^of water.^19^ According to the absorption model described by Silverstein et al., the superabsorption of PE-PHs is related to their unique microstructure consisting of quasi-spherical voids connected with numerous smaller circular holes called windows, where the voids are initially filled through the capillary action and absorption then continues at the expense of the hydrogel-swelling-driven void expansion mechanism.^4,11^ In addition to high absorption capacity, some of the PE-PHs also exhibited exceptional water retention properties under compression (up to 60% strains) and then even recovered to their original heights upon the removal of stress without failing.^4^ Due to these excellent properties, the potential applications of these PE-PHs are therefore wide-ranging and include environmental applications for removing contaminants from water,^20^ fire-retardant materials,^7^ desiccants for organic solvents,^16^ or scaffolds for tissue engineering applications.^21^ Despite their unique water absorption/retention properties, the use of PE-PHs in agriculture and horticulture is surprisingly low, although these materials could ease the burden of water shortage in a dry soil. Akay et al. reported the first use of sulfonated PHs in an agro-process intensification application as a soil conditioner to improve hydrological properties,^22−24^ but apart from these examples, the application of PHs in agriculture and horticulture has not received much attention. Moreover, the use of PE-PHs as substrates for in vitro germination and seedling growth via the soilless cultivation technique is completely unexplored.

The soilless cultivation technique, which uses synthetic substrates instead of soil as rooting medium, is a system for in vitro plant growth.^25^ Because traditional soil-grown cultivation cannot keep up with the ever-increasing global demands, in vitro culture techniques are now indispensable. Embryo culture, a type of plant tissue culture, is an important in vitro technique in which the plant develops directly from the embryo on a growth substrate.^26,27^ It is an effective technique that shortens the reproductive cycle of plants by growing excised embryos, reducing the long dormancy of seeds and accelerating plant development.^28^ In this process, the growing substrate is extremely important. It can be either inert organic or inorganic growing media filled with a nutrient solution.^29^ An effective substrate must have appropriate physical and chemical properties, such as (i) a uniform microstructure that drains well, but retains nutrients and water for the root system, (ii) adequate bulk density/porosity to provide space for root development and facilitate the transportation of water/nutrients, and (iii) chemical inertness.^29^ Also, very important is the ability to retain original characteristics, so that it can be reused in many successive growing cycles.^30^ Currently, agar is still the most commonly used growth medium for in vitro cultivation. However, the major disadvantages of agar-based growth substrates are low water/nutrient diffusion, low oxygen concentration, low mechanical resistance, and inability to maintain initial properties when reused. Therefore, among growth media, covalently cross-linked hydrogels have proven to be good soilless cultivation substrates due to their robustness and reserved water availability.^31^ However, introducing porosity into the hydrogel network, for example, as in PE-PHs, would allow more water/nutrients to be stored on the one hand and it will improve transport within the hydrogel porous structure on the other, allowing gradual release to the plant root system. In this way, the supply of water/nutrients becomes more efficient, which will greatly affect the germination of seeds or seedlings to grow over a long period of time.

Considering the ongoing expansion of soilless cultivation, the development of this technique in the future largely depends on the design of an optimal substrate, as this is determinant for the survival of plants grown in vitro. An optimal substrate that takes into account physical, chemical, and environmental factors has yet to be developed. Therefore, in this work, we explore the great potential of highly porous, 3D interconnected microcellular PE-PHs as substrates for soilless cultivation, which to our knowledge have not been used before. The isolated embryo axes of chickpea seeds were inoculated onto the surface of the swollen and autoclaved PE-PHs and germinated. The interactions between the embryo axes and PE-PHs were studied and productivity evaluated in terms of the number of shoots, roots and leaves developed, and the length of shoots and roots compared to agar as the standard substrate.

## 2 Experimental Section

### Materials

(3-Acrylamidopropyl)-trimethylammonium chloride (AMPTMA, 75 wt % in H_2_O, Sigma-Aldrich); methylene bis-acrylamide (MBAAm, Sigma-Aldrich); poly(ethylene oxide)-*block*-poly(propylene oxide)-*block*-poly(ethylene oxide), MW = 12,600 g·mol^–1^; the so-called Pluronic F-127, Sigma-Aldrich; ammonium persulfate (APS, Fluka); *N*,*N*,*N′*,*N′*-tetramethylethylenediamine (TMEDA, Sigma-Aldrich); ethanol (Sigma-Aldrich); diethyl ether (Merck); and toluene (Merck) were all used as received. Embryo axes of chickpea (*Cicer arietinum* L.) were used as initial explants (see the Supporting Information).

### Preparation of AMPTMA-Based PE-PH from O/W HIPE

AMPTMA O/W HIPE and polyHIPE thereof was prepared according to the method and technique published elsewhere.^5^ Briefly, water (5 mL), AMPTMA (2.51 g), MBAAm (0.15 g), Pluronic F-108 (0.4 g), and APS (0.1 g) were placed in a three-necked 250 mL flask and the mixture was stirred with an overhead stirrer at 400 rpm. Then, the corresponding amount of toluene (20 mL) was added dropwise under constant stirring and once all toluene had been added, stirring was continued for further 10 min to produce the uniform O/W emulsion. Afterward, the reducing agent TMEDA (80 μL) was added during reduced stirring (20 rpm) and the emulsion transferred to the mold and cured for 24 h at 40 °C. The resulting polyHIPE monolith was purified via Soxhlet extraction with ethanol and ether, each for 24 h and then vacuum-dried until constant weight was attained.

### Culture Conditions and Germination

Details on embryo axes isolation and substrate preparation are described in the Supporting Information. Briefly, chickpea seeds were sterilized with 1% NaOCl for 10 min and rinsed three times with sterile water before the embryo axes were aseptically separated from the cotyledon tissue (Figure S1) and immediately inoculated on two different substrates, agar and PE-PH. Prior to inoculation, the PE-PH substrate was immersed in deionized water and then exchanged with the MS medium containing 3% sucrose (see the Supporting Information). In the first experiment, 42 embryo chickpea axes were aseptically inoculated onto the surface of agar and PE-PH substrates to determine their growth and development into shoots and roots. Shoot and root formation was determined by measuring the length of shoots and roots every week for 4 weeks (Figure S2). In the second experiment, 12 embryo chickpea axes were inoculated onto the surface of agar and PE-PH substrates to determine the fresh weight (FW) and dry weight (DW) of developing shoots and roots after 1 (t1), 5 (t2), 8 (t3), 11 (t4), and 15 (t5) days, for a total of 144 embryo axes in both experiments. The third experiment determined the effect of chickpea seed germination on the reused PE-PH substrate. For this purpose, the same PE-PH substrate was reused five times and embryo chickpea axes were inoculated. All experiments were repeated twice and four replicates of each experiment. All data were finally statistically analyzed (seethe Supporting Information).

### Characterization

Chemical structure was characterized by Fourier transform infrared (FTIR) spectroscopy. FTIR spectra were recorded on a PerkinElmer Spectrum One instrument (PerkinElmer, Inc., Waltham, MA, USA). Elemental analyses were performed to determine the nitrogen content in the resulting polyHIPEs (Flash 2000 CHNS Analyzer, Thermo Scientific). Porous structure of the dry polyHIPEs was studied by scanning electron microscopy (SEM) (Carl Zeiss, SUPRA 35 VP microscope). A piece of each sample was cryogenically fractured and mounted on a carbon tab for better conductivity. A thin layer of gold was sputtered on the sample’s surface prior to SEM analysis. The polyHIPE densities (ρ_PH_) were determined gravimetrically. The polyHIPE skeletal (polymer wall) densities (ρ_P_) was analyzed using a fully automated, high-precision helium pycnometer (Micromeritics AccuPyc II 1340).

## 3 Results and Discussion

**Figure 1:**
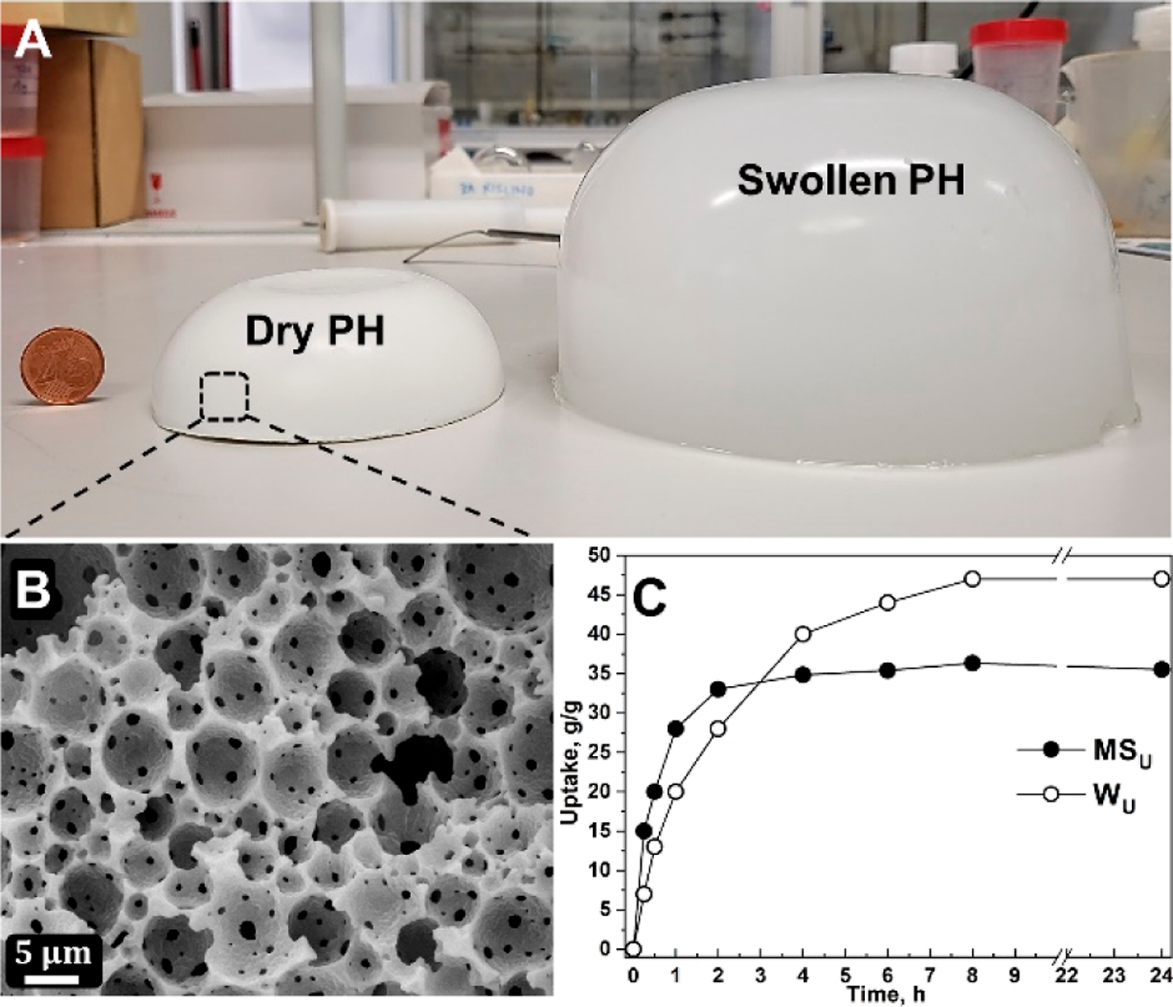
Dry and equilibrium water swollen AMPTMA-PH (A); scanning electron micrographs of dry PH (B); and uptake as a function of time (C).

The highly porous PE-PH substrates were successfully synthesized through the O/W HIPE templating. HIPEs were obtained using two immiscible liquids, toluene as the pore templating phase and water as the polymerization phase, in which AMPTMA and MBAAm were dissolved as monomers along with the water-soluble redox couple APS/TMEDA for rapid polymerization at room temperature. The as-synthesized polyHIPEs were, after purification and drying, white monoliths (Figure 1A left) with densities of ∼0.27 g·cm^–3^ and porosities of ∼77%. The porous structure is seen in Figure 1B. The size of the voids (primary level pores) is 5 ± 3 μm whereas the size of the interconnecting pores (secondary level pores) is about 1 μm, resembling a typical highly interconnected, open porous PH structure. Chemical characterization of PE-PH included elemental analysis and FTIR spectroscopy. Both confirmed the presence of AMPTMA cross-linked with MBAAm in the polymer network. FTIR spectra obtained from different positions of a freshly cut monolithic sample immediately after purification and drying contain typical peaks at 1480 and 960 cm^–1^ corresponding to CH_3_ stretching and bending vibrations associated with the −N^+^(CH_3_)_3_ group in AMPTMA, respectively, whereas peaks at 1640, 1530, and 1120 cm^–1^ indicate vibrations of the amide C=O (amide I), N–H bending, and C–N stretching, respectively (Figure S3). The elemental composition of the AMPTMA-based PE-PH foam studied by elemental analysis revealed a high nitrogen content, which was 11.9 wt % N, 44 wt % C, and 9.5 wt % H, corresponding to 8.51 mmol nitrogen (3.83 mmol of −NR_3_^+^ groups) per gram of PH.

Another distinctive feature of PE-PHs is their extraordinary capacity for water absorption and good monolithic robustness in the swollen state (Figure 1A), a very important property when such materials are envisaged as substrates for germination and subsequent seedling as shown herein. The extent of swelling ratio (*S*_R_) and equilibrium water (*W*_U_) or MS medium uptake (MS_U_) as a function of time was further investigated. The dried monoliths began to swell immediately upon contact with water or MS medium to larger sizes. The AMPTMA-based PE-PH monoliths show *S*_R_ of 8 or 6 cm^3^·cm^–3^ for water or MS-medium, respectively. The equilibrium absorption of water and MS medium for the AMPTMA-based PE-PH is shown in Figure 1C with *W*_U_ of 42 g·g^–1^ and MS_U_ of 36 g·g^–1^, respectively. Both *S*_R_ and the total absorption capacity for MS medium are similarly high to that of water. The rapid swelling and thus absorption capacity of the AMPTMA-based PE-PH monoliths is also impressive, reaching equilibrium values between 2 to 8 h (Figure 1C). The rationale for this fast swelling behavior and high absorption capacity is the result of our material design, which combines polyelectrolyte properties, that is, a polymer network with charged quaternary nitrogen groups (−NR_3_^+^) and a highly porous 3D interconnected structure that forms extensive capillary channels, which help the dried polymer start to swell within minutes.

**Figure 2:**
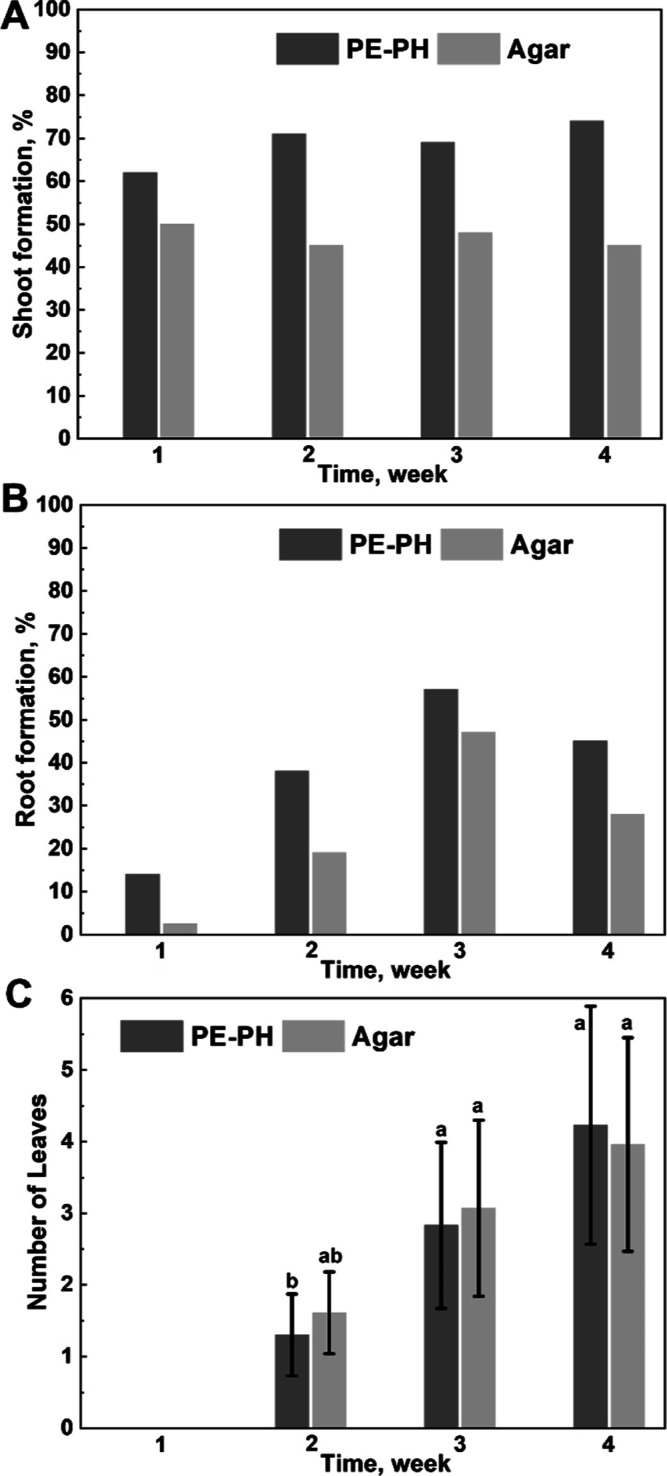
Shoot formation percentage (A); root formation percentage (B); and number of leaves (C) of chickpea embryo axes (ANOVA, Kruskal–Wallis Test).

When the PH substrate is used for germination and seedlings, such a foamy structure should be advantageous, for example, for the association of roots that not only adhere to the polymer surface but have also the possibility to grow through the structure, making the PH substrate an integral part of the root network. However, the foamy structure is not the only important feature of the synthetic PH substrate, but its chemistry must also be compatible with the seeds during germination. To investigate whether the synthesized AMPTMA-based PE-PH can affect the germination and development of shoots and roots, the embryo axes of chickpea seeds were inoculated onto the surface of MS swollen and autoclaved PE-PH substrate. In parallel, germination was also performed on standard growth medium, that is, agar (Figure S2). It was found that 62% of embryo axes developed shoots in the first week, and this number increased steadily with time, reaching 74% in the fourth week of cultivation (Figure 2A). On the other hand, shoot formation on agar was not as efficient, with only between 45 and 50% of embryo axes developing shoots during the 4 week period, indicating that shoot formation was significantly higher on the PH substrate than on agar. The length of the shoots developed on the PE-PH substrate was slightly longer than those on agar. They grew to 1 cm in length within the first week and developed between 4 and 5 cm in length by the end of the fourth week (Table 1). After the first week, about 11 or 2% of the embryo axes germinated on the PE-PH substrate or on the agar substrate, respectively, also developed roots. The number of roots then continued to increase and was the highest in the third week on the PE-PH substrate at 57%, whereas by the fourth week on agar it was 45% (Figure 2B). There were no significant differences between the length of the roots grown on the PE-PH or agar substrate and were about 1, 4, or 5 cm long after the second, third, and fourth week, respectively (Table 1). Finally, leaves began to develop after the second week, starting with about one leaf and ending with three leaves after the fourth week per plantlet.

**Table 1:** Overall Mean Values for Different Characteristics of Chickpea Embryo Axes Grown on Different Substrates

	PH substrate				agar substrate			
parameters	W1	W2	W3	W4	W1	W2	W3	W4
embryo axes^a^	42	42	42	42	42	42	42	42
no. of shoots	26	30	29	31	21	19	20	19
shoot length, [cm]	1.2 ± 0.4	3.2 ± 1.2	5.3 ± 2.1	5.9 ± 2.3	1.1 ± 0.3	2.8 ± 0.9	4.3 ± 1.7	5.0 ± 2.1
no. of roots	6	16	24	19	1	8	20	12
root length, [cm]	0.6 ± 0.2	3.3 ± 1.2	3.7 ± 1.8	5.5 ± 2.5	0.3 ± 0.0	3.4 ± 1.1	4.2 ± 2.7	9.3 ± 3.1
no. of leaves		1.9 ± 0.6	2.9 ± 1.2	5.3 ± 1.7		1.9 ± 0.6	3.1 ± 1.2	4.7 ± 1.5
hyperhydration^b^		13	16	13		3	4	3

a Initial number of inoculated embryo axes.

b Number of plants with hyperhydration.

**Figure 3:**
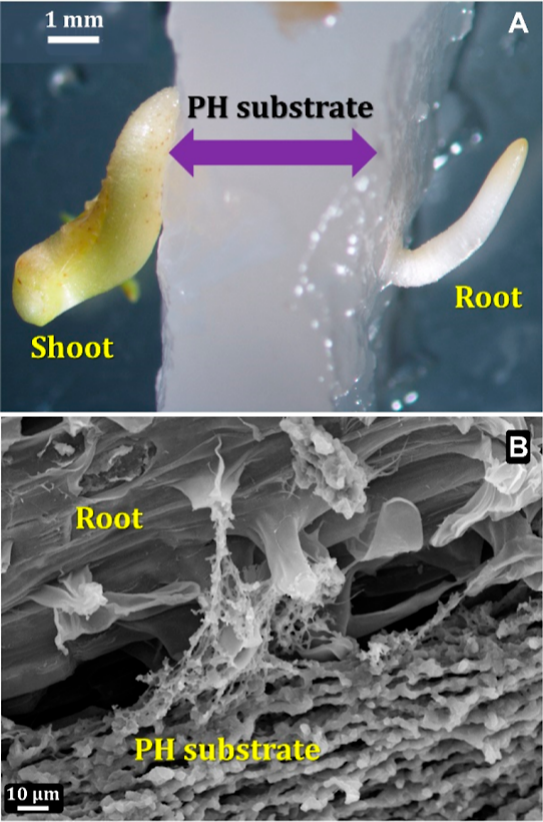
Optical micrograph of chickpea root penetrating the PH substrate (A) and SEM image of the root associated with the foamy PH structure (B).

Next, the influence of substrates on the FW and DW of the developed shoots and roots during the 15 day period of development was studied, which in principle reflects the ability of the plant to continue growing (Figure S4). The FW of shoot and root was almost the same in both substrates after the first day of cultivation and then increased gradually to their final mass, which doubled after 15 days. Only small, non-significant differences in the increase of FW were observed between the substrates. The gradual increase in mass was also observed in DW for both shoots and roots, with the significant difference observed in the higher DW of shoots after 15 days of cultivation on agar (Figure S4). Interestingly, despite the better shoot formation, the DW was lower for those that grew on the PH substrate than in those that grew on agar, indicating hyperhydration (HH) symptoms in the case of the PE-PH substrate. Hyperhydration (HH) or vitrification is a physiological disorder that often affects vegetatively propagated shoots in vitro and is usually due to higher water availability in the substrate or higher relative humidity in the confined atmosphere of the flask.^32,33^ In our case, hyperhydric plants had light green stem and thicker, translucent shoots. The abnormality occurred in 13 cases after 2 weeks (31%) and then increased to 16 after 3 weeks (38%), whereas on agar only about 3–4 cases (∼8%) developed HH symptoms (Table 1). We believe that HH was due to the large amount of MS medium stored in the highly macroporous structure, as the absorption capacity of the PE-PH substrate of 36 g·g^–1^ is significantly higher than that of agar (20 g·g^–1^). The macroporous structure also stimulates roots to grow inside the PH substrate rather than just adhering to the surface. About 14% of such embryo axes forming roots penetrating the substrate were found (Figure 3A), with the substrate being an integral part of the root network (Figure 3B).

Finally, we investigated the germination of chickpea embryo axes on a different PH substrate, namely an anionic polyelectrolyte network containing negatively charged sulfonate groups (−SO_3_^–^) such as PAMPS.^4^ A clear difference was observed compared to a cationic AMPTMA-based PE-PH substrate, as germination was completely inhibited and neither shoots nor roots were seen after 4 weeks of cultivation. In the end, the reusability of the AMPTMA-based PE-PH substrate was investigated. The PE-PH substrate was Soxhlet-extracted overnight in ethanol after initial use and immersed in water for solvent exchange. After several ethanol/water exchange cycles, the substrate was soaked in MS medium, autoclaved (at 121 °C and 1.2 bar for 15 min), and reused. One set of the already used PE-PH substrates was not autoclaved after soaking in MS medium. The embryo axes of the chickpea seeds were then inoculated onto the surface of the autoclaved and non-autoclaved PE-PH substrates and surprisingly, germination was successful on both. After five consecutive reuses (Soxhlet extraction/MS soaking/autoclaving), no damages were observed to the AMPTMA-based PE-PH monoliths, indicating a very robust synthetic substrate.

## 4 Conclusions

In summary, we have presented the idea of using PE-PHs as substrates for soilless cultivation of plants. The results indicate that the AMPTMA-based PE-PH substrate apparently combines advantageous properties such as a well-developed macroporous structure, suitable mechanical properties, and appropriate chemistry, all of which together promote efficient germination of chickpea seed embryo axes. In fact, the PE-PH substrate performed better than agar in all respects when comparing the number of shoots germinated or roots developed, the length of shoots and roots, or the number of leaves developed during the 4 week culture. In addition, the PE-PHs are reusable substrate and can be used even without prior autoclaving (sterilization). The only drawback we observed with the PE-PH substrate was that a small proportion of plants (∼35%) developed HH symptoms. However, the latter can be overcome by adjusting the physiochemical properties of the PE-PH substrate and is currently under investigation in our laboratories. We believe that the combination of facile synthesis, scalability, and reusability of polyelectrolyte polyHIPEs is very advantageous compared to the commonly used agar, both from an economic and sustainability point of view, and that the results described here can provide a great incentive for further exploration of polyHIPEs as substrates in agriculture and horticulture for soilless cultivation.

## Supporting Information Available

Details on embryo axes isolation and substrate preparation, FTIR spectrum, and data of the FW and DW of developed shoots and roots with corresponding statistics (PDF)
