# Supplementary material for: Use of Emulsion-Templated, Highly Porous Polyelectrolytes for In Vitro Germination of Chickpea Embryos: a New Substrate for Soilless Cultivation
Source: Biomacromolecules. 2022 Jul 8;23(8):3452–7. doi: 10.1021/acs.biomac.2c00593 (PMC9364313; doi:10.1021/acs.biomac.2c00593)
Supplement: Supplementary file 1 — bm2c00593_si_001.pdf [file bm2c00593_si_001.pdf]

The use of Emulsion-Templated, Highly-Porous Polyelectrolytes for *in vitro* Germination of Chickpea Embryos: A New Substrate for Soilless Cultivation

Janja Majer Kovačič,[a] Terezija Ciringer,[a] Jana Ambrožič-Dolinšek,[a,b,c] Sebastijan Kovačič[d]*

[a]Faculty of Natural Sciences and Mathematics, University of Maribor, Koroška 160, 2000 Maribor, Slovenia.

[b]University of Maribor, Faculty of education, Koroška cesta 160, 2000 Maribor, Slovenia

[c]University of Maribor, Faculty of Agriculture and Life Sciences, Pivola 10, 2311 Hoče, Slovenia

[d]National Institute of Chemistry, Department of Polymer Chemistry and Technology, Hajdrihova 19, 1000 Ljubljana, Slovenia. E-mail: sebastijan.kovacic@ki.si

Supplementary Information

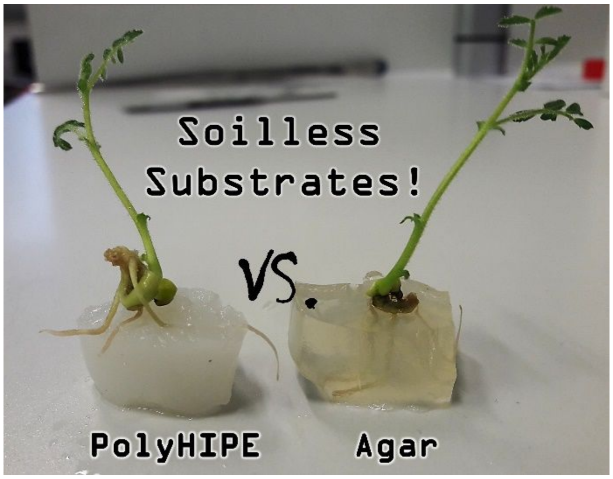

1. Experimental

Culture conditions and germination. First, seeds of chickpea were soaked in 1% commercial bleach (<5% NaOCl), with a drop of the detergent Tween 80 for 10 min, rinsed three times with sterile deionized water, and immersed them for 24 h in sterile deionized water for imbibition. Embryonic axes (Figure S1) were then aseptically separated from cotyledon tissue and used immediate inoculated on two different substrates, agar and PE-PHs. Both substrates were first soaked with Murashige and Skoog (MS) (1962) liquid medium and 3% sucrose. The agar substrate was soaked *in situ*, i.e., 0.8 % of Difco bacto-agar medium was mixed with the liquid MS medium and 3% sucrose and allowed to solidify. For the PE-PH substrates, the preformed PH monoliths were first soaked with deionized water, followed by the MS medium and 3% sucrose. Vessels containing MS medium-soaked agar and PE-PH substrates were then autoclaved at 121° C and 1.2 bar for 15 minutes. Before autoclaving, the pH was adjusted to 5.7-5.8. Embryonic axes were then cultured on substrates containing 20 mL of liquid MS medium maintained in a growth chamber at 23 ± 2°C with Osram L 36 W/77 FLUORA fluorescent tubes at a 16-hour photoperiod and a light intensity of 37-50 μmol $m^{-2} \cdot s^{-1}$.

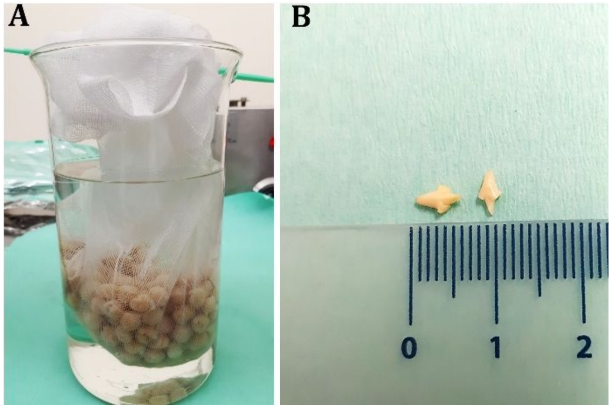

Figure S1. Sterile chickpea seeds (**A**) and aseptically separated embryo axes (**B**)

Statistical analysis. The statistical software package SPSS® 27.0 (SPSS Inc., Chicago, IL) was used for data analysis. The analysis of variance (ANOVA) using the post hoc Duncan test and for nonparametric Kruskal-Wallis Test were used for evaluating the levels of statistical significance (P) between the control samples on agar and the PE-PH substrate. The significant differences were indicated by distinct letters as in Figure 2C in Tables and Figures. The symbols used in the figures, e.g., 2C are as follows: NS denotes not significant, * denotes $P < 0.05$, ** denotes $P < 0.01$, *** denotes $P < 0.001$. All experiments were repeated at least twice. In each repetition, at least four replicates, with no fewer than 12 explants per replicate, were used.

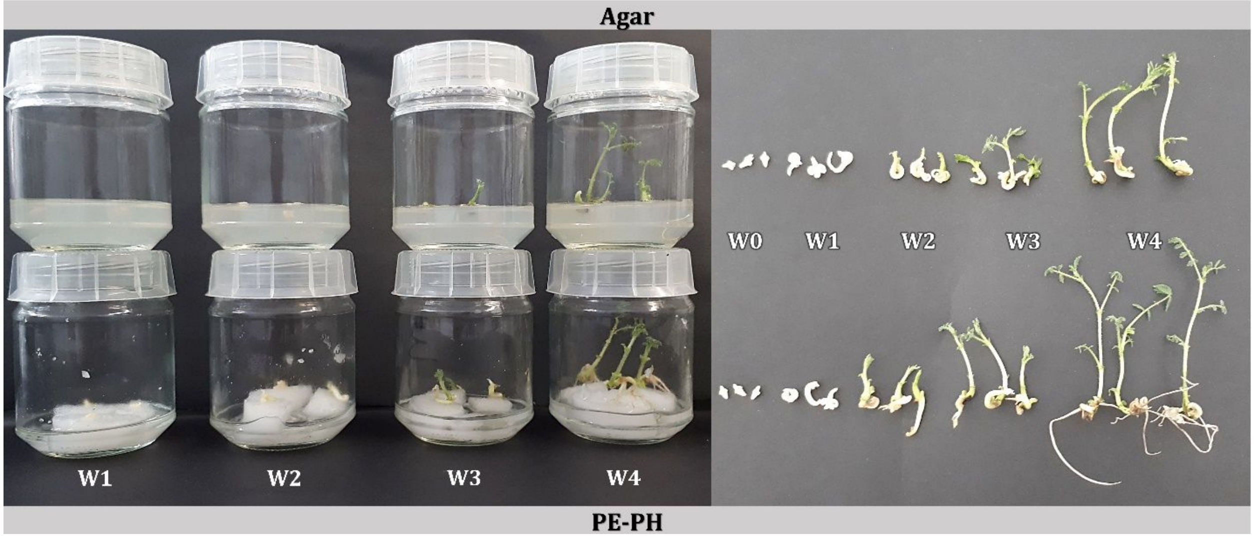

Figure S2. Development of chickpea after embryonic axes inoculation (left) and *in vitro* raised seedlings after single week (right) over a period of four-weeks.

2. Characterization

FTIR Spectroscopy of AMPTMA-based polyHIPE

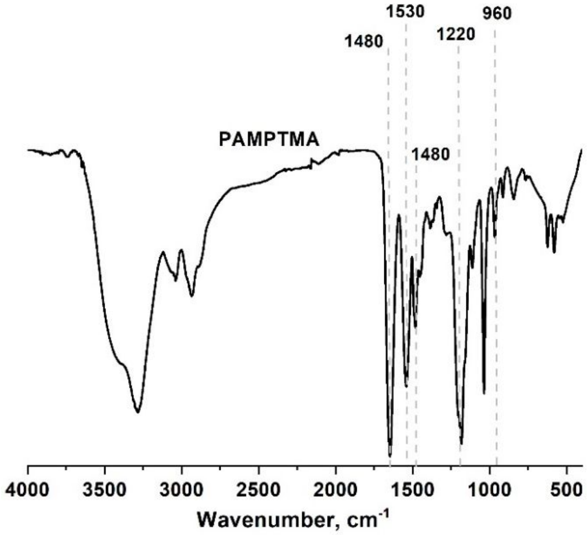

Figure S3. FT-IR spectra of AMPTMA-based PH substrate

The influence of substrates on the FW and DW of developed shoots and roots

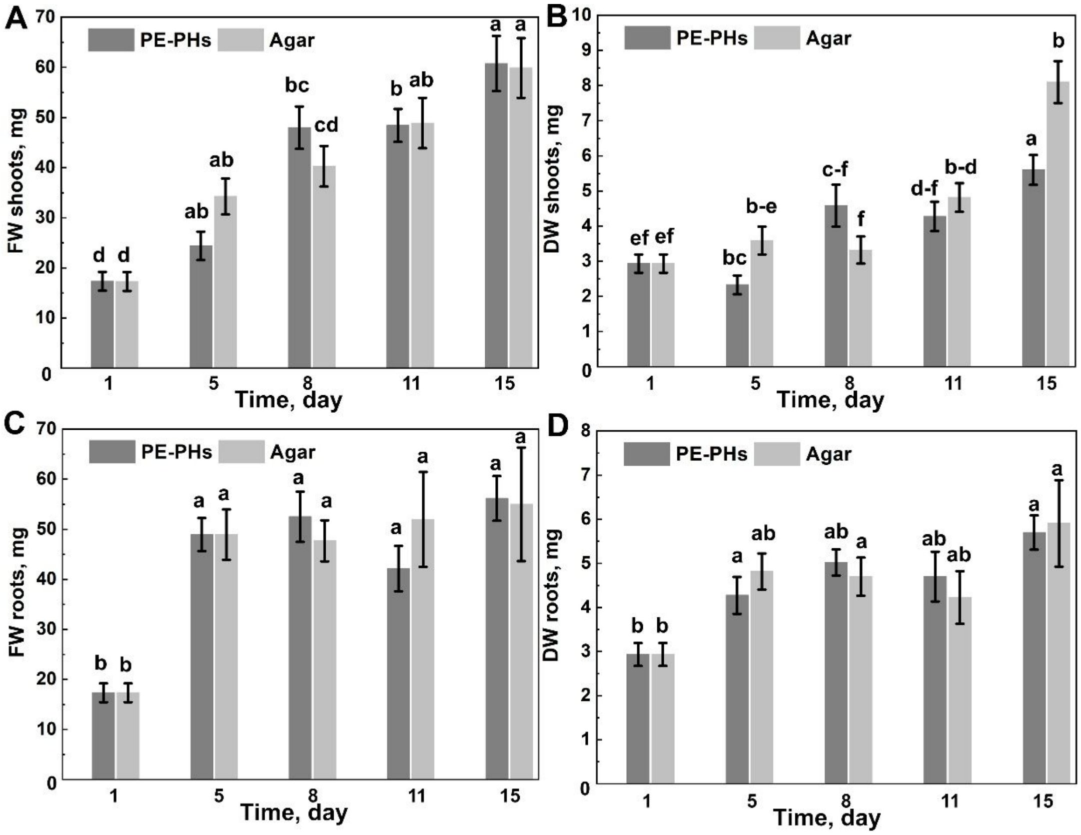

Figure S4. Fresh weight (A and C) and dry weight (B and D) (n = 15) within the 15 days of germination comparatively on PE-PH and agar substrates (ANOVA, Duncan test).
